# Diet and asthma: looking back, moving forward

**DOI:** 10.1186/1465-9921-10-49

**Published:** 2009-06-12

**Authors:** June-Ho Kim, Philippa E Ellwood, M Innes Asher

**Affiliations:** 1 Department of Cancer Immunology & AIDS, Dana-Farber Cancer Institute, Boston, Massachusetts, USA; 2 Department of Paediatrics: Child and Youth Health, The University of Auckland, New Zealand

## Abstract

Asthma is an increasing global health burden, especially in the western world. Public health interventions are sought to lessen its prevalence or severity, and diet and nutrition have been identified as potential factors. With rapid changes in diet being one of the hallmarks of westernization, nutrition may play a key role in affecting the complex genetics and developmental pathophysiology of asthma. The present review investigates hypotheses about hygiene, antioxidants, lipids and other nutrients, food types and dietary patterns, breastfeeding, probiotics and intestinal microbiota, vitamin D, maternal diet, and genetics. Early hypotheses analyzed population level trends and focused on major dietary factors such as antioxidants and lipids. More recently, larger dietary patterns beyond individual nutrients have been investigated such as obesity, fast foods, and the Mediterranean diet. Despite some promising hypotheses and findings, there has been no conclusive evidence about the role of specific nutrients, food types, or dietary patterns past early childhood on asthma prevalence. However, diet has been linked to the development of the fetus and child. Breastfeeding provides immunological protection when the infant's immune system is immature and a modest protective effect against wheeze in early childhood. Moreover, maternal diet may be a significant factor in the development of the fetal airway and immune system. As asthma is a complex disease of gene-environment interactions, maternal diet may play an epigenetic role in sensitizing fetal airways to respond abnormally to environmental insults. Recent hypotheses show promise in a biological approach in which the effects of dietary factors on individual physiology and immunology are analyzed before expansion into larger population studies. Thus, collaboration is required by various groups in studying this enigma from epidemiologists to geneticists to immunologists. It is now apparent that this multidisciplinary approach is required to move forward and understand the complexity of the interaction of dietary factors and asthma.

## Introduction

Asthma, particularly among children, has grown in prevalence and as a worldwide public health burden [1], but has been an elusive target for public health interventions. Dietary factors have been a focus at both the cellular and population levels, and several theories have been proposed or abandoned, though no clear answer has emerged [2-12]. This review highlights the development of major promising hypotheses about diet and asthma and possible paths for future investigation.

## Nature to nurture

Asthma is an allergic disease of complex gene-environment interactions [13-15]. Twin studies show that over 70% of the variation in asthmatic tendency is explained by genetic factors, and several contributing genes have been identified [16,17]. However, individual genes have been ineffective in altering the expression of asthma, indicating the necessity of environmental factors [14]. Rapid increases in worldwide asthma prevalence in only the past couple decades, especially in westernized countries, signal an important role of the environment [12].

It is known that environmental factors affect gene expression and manifestation of disease. Early fetal exposures to nutrition and other environmental factors may program organ development and future development of disease. For example, severe fetal malnutrition has been linked to increased risk for health problems in adulthood [18]. Thus, nutrition and diet may be important to the development of asthma through epigenetic effects. With rapid changes in diet as a hallmark of westernization, dietary factors may indeed play a key role in affecting the complex genetics and developmental pathophysiology of asthma.

## Early dietary hypotheses

It is important to look back on the progression of dietary studies over the years to see how theories have evolved and adapted as new evidence has been brought forth and new ideas proposed.

### Hygiene hypothesis

Increased westernization and the correlated rise in asthma prevalence have prompted investigation of environmental factors related to westernization. One of the earliest theories became known as the "hygiene hypothesis," which suggested that increasing "cleanliness" and lack of exposure to infections at a critical point in the development of the immune system may lead to an increased risk of asthma and other atopic diseases [19]. This hypothesis has not been well supported by evidence, such as an increase of asthma in North and South American inner cities that are generally characterized by poor housing and a dirty environment [12,20,21].

### Antioxidant hypothesis

Seaton et al. 1994 hypothesized that alteration in diet associated with westernization may be responsible for the increase in asthma prevalence [22]. Observations showed that consumption of foods rich in antioxidants had decreased in the United Kingdom diet while asthma prevalence rose. Thus the promising hypothesis was put forth that populations had become more susceptible to respiratory disease due to dietary antioxidant omission.

Antioxidant studies have focused on vitamin C, vitamin E, carotenoids, flavonoids, and antioxidant nutrients such as selenium and zinc. A wide range of cross-sectional studies has been done on the relationship of antioxidants with asthma. Vitamin C, β-carotene, magnesium, and selenium were associated with reduction in asthma prevalence [23-27], and may prevent or limit an inflammatory response in the airways by reducing reactive oxygen species and inhibiting lipid peroxidation. Flavonoids may also be potential anti-allergic substances [28], and a recent study on enzymatic and nonenzymatic antioxidant systems in childhood asthma suggested that antioxidant defenses such as glutathione peroxidase and superoxide dismutase were lowered in asthmatic children [29].

However, not all studies on the role of antioxidants have been positive. A meta-analysis determined that dietary intake of antioxidants vitamins C and E and β-carotene does not significantly influence the risk of asthma [30]. Furthermore, many studies have shown no association between selenium and asthma [31]. However, these results may still have significance in light of biological studies that show that selenium acts as an antioxidant but can also upregulate immune responses that characterize allergic asthma – a more complex effect that cannot be explained just by case-control studies [32]. The potential role of antioxidants as supplements has been explored [33], but a number of studies have been inconclusive [34]. Overall, supplementation studies have suggested a minor role for individual antioxidants in asthma prevention [4], perhaps working in larger food groups instead – the source of Seaton's original study.

### Lipid hypothesis

In 1997, Black and Sharpe cited evidence, which contradicted the antioxidant hypothesis, instead proposing that the rise of asthma prevalence may have stemmed from increased consumption of polyunsaturated fatty acids (PUFAs) and decreased consumption of saturated fat [35]. The ω-6 PUFAs may particularly have a role in regulating immune response and inflammation. These PUFAs are found largely as linoleic acid in foods such as margarine and vegetable oils, which have risen in consumption with westernization. Linoleic acid is a precursor of arachidonic acid that is converted into prostaglandin E_2 _(PGE_2_), which inhibits interferon-γ (IFN-γ) and promotes an inflammatory environment that favors asthma development. Meanwhile, ω-3 PUFAs may have an anti-inflammatory role. Thus, the increase in ω-6 PUFA and decrease in ω-3 PUFA consumption may immunologically increase the susceptibility of the population. PUFAs may have other immunosuppressive mechanisms that require further study [36].

Investigation of the lipid hypothesis found mixed results. A number of cross-sectional studies showed beneficial associations between foods containing ω-3 PUFAs and asthma, but studies on cord blood PUFA composition and development of atopic disease have been inconclusive [5]. There have been conflicting reports on the relationship between levels of PUFAs and wheeze [37,38]. Disappointingly, intervention studies have not found consistent results nor provided sufficient support for dietary supplementation with PUFAs [36,37,39-41].

### Other nutrients

Other nutritional factors have recently been investigated using various methods ranging from cohort studies to ecological analyses with populations from schoolchildren to entire nations.

A sodium hypothesis was proposed in 1987 based on a correlation between table salt purchases and asthma mortality [42]. Sodium intake could potentially exacerbate asthma as hyper-sensitized bronchial smooth muscle could be leaky to sodium and thus lead to hyperpolarization of the muscle in response to increased sodium intake [43]. However, there is no clear relationship between airway responsiveness (a surrogate for asthma) and urinary sodium excretion (an indicator of sodium intake) [44]. A more recent trial, in which participants adopted a variable sodium diet based on supplements or placebo, found no benefit for asthma either [45].

Magnesium has been implicated through its possible effects on bronchial smooth muscle. Low magnesium intake has been correlated with decreased lung function in children [46], and intravenous magnesium is recommended to control acute severe asthma in many emergency departments [47]. Nevertheless, due to a paucity of studies on magnesium and asthma prevalence, its importance remains to be seen.

### Food types and dietary patterns

Larger food groups have been studied as possible examples of synergy among multiple nutrients. Fruits and vegetables have been extensively studied as potent sources of antioxidants. A low dietary intake of fruit was associated with asthma in Norwich, UK [25]. Several other cross-sectional studies have indicated an inverse association between consumption of fruits and vegetables and symptoms of asthma, though the particular foods and symptoms varied [8,48-52]. Moving beyond individual country studies, Ellwood et al. conducted an ecological analysis on data from centers in 53 countries the International Study of Asthma and Allergies in Childhood (ISAAC), which not only looked at single countries, but also compared diet and asthma globally using asthma prevalence data from ISAAC and dietary data from the Food and Agriculture Organization of the United Nations [53]. Together, these data suggested an inverse relationship between asthma prevalence rates and intake of vegetables and foods of plant origin such as starch and cereals. However, a smaller study of Dutch children found no clear association between fruit and vegetable intake and asthma symptoms [54]. Despite the plethora of cross-sectional data about fruits and vegetables, there is a lack of longitudinal studies and analyses to form a causal link between these foods and asthma prevalence.

The hypothesis of westernized diets affecting asthma prevalence has prompted studies of fast foods, Mediterranean diet, and obesity as potential factors. A cross-sectional study of children in Hastings, New Zealand showed that hamburger consumption positively associated with asthma symptoms while takeaway consumption had a marginal effect on bronchial hyperresponsiveness [55].

The Mediterranean diet, on the other hand, has been suggested as a healthy dietary pattern that may reduce the risk of asthma. In fact, ISAAC data indicated lower asthma prevalence in Mediterranean countries with diet as a possible variable to explain this disparity [1,56,57]. There is a consistent relationship between a Mediterranean diet and asthma symptoms [48,57,58]. But additional studies are necessary to corroborate this association and define a possible mechanism.

Lastly, obesity is a major factor of diet that may have a role in asthma. Its role has been controversial as, yet again, different studies have found contrasting results [58]. Epidemiologic studies have suggested that asthma is more prevalent among obese than lean individuals. It is unclear, however, whether obesity merely exacerbates the asthmatic symptoms, creates susceptibility to onset of asthma, or develops concurrently with the respiratory disease. Obesity could have potential biological effects on lung function and systematic inflammation while also sharing certain co-morbidities and etiologies with asthma [59]. Nevertheless, the relationship between obesity and asthma remains an enigma despite evidence of a connection.

Overall, interesting hypotheses and some promising positive findings have made no definitive conclusions about the role of specific nutrients, food types, or dietary patterns on asthma prevalence.

## Evolution of dietary hypotheses and studies

Recent work has linked diet to the development of the fetus and child – an extrapolation from studies on other diseases indicating an effect of early diet on later onset of disease. This "thrifty phenotype hypothesis" argues that poor nutrition in early life is epidemiologically associated with poor fetal and infant growth and subsequent development of type 2 diabetes [60]. A large body of evidence shows that the intrauterine and early childhood environments are crucial for development of diabetes and coronary heart disease, and asthma has been increasingly included in a similar category of diseases "programmed" in utero [61], hinting at a possible epigenetic component. This developmental model of the origins of disease possesses a variety of subcategories that have been recently explored for asthma from breastfeeding and intestinal microbiota to maternal nutrition.

### Breastfeeding

Breastfeeding provides infants with nutrients for growth, development, and immunological protection during a critical period of the infant's life when its own immune system is immature [62,63]. There are many questions about exclusive breastfeeding over infant formula and the optimal length of breastfeeding in asthma development. A 2004 cohort study showed exclusive breastfeeding for more than four months reduced the risk of asthma at the child's age of four [64]. A separate 2008 cohort report on the Avon Longitudinal Study of Parents and Children (ALSPAC) agrees that breastfeeding has a modest protective effect against wheeze and asthma in early childhood [65]. However, the study found that this effect did not last beyond the sixth year of life. Despite some positive studies, others have seen an entirely converse effect [66], leading to some heated controversy about breastfeeding recommendations [67,68].

Breastfeeding is complex in its effects on the immunological health of the child. Regardless, not enough evidence exists to recommend guidelines for breastfeeding for asthma prevention.

### Probiotics and intestinal microbiota

Breastfeeding is well known to modify the intestinal composition of commensal bacteria, which drives immune development in the infant. For example, exclusively formula-fed infants possessed more colonies of *E coli*, *C difficile, Bacteroides*, and lactobacilli compared to breastfed infants [69]. Instead, breastfed infants had the most potentially beneficial intestinal microbiota. The human gastrointestinal tract is sterile at birth, rapidly undergoing colonization of the gut with subsequent development of the immune system. Studies have shown that there are obvious differences in the composition of intestinal microbiota between healthy and allergic infants within the first week of life and before clinical symptoms for the latter group, suggesting that modifying microbiota composition may affect disease outcome [70].

Probiotics are dietary supplements that contain beneficial bacteria such as *Lactobacillus GG *and may be effective in preventing early atopy in children through the modulation of intestinal microbiota [71]. Probiotics may enhance IgA responses in the gut as well as regulate inflammatory cytokines, both immunomodulatory effects that could prevent progression of atopy and potentially development of disease. Further study, possibly large-scale birth cohort analyses using molecular methods to test for microbiota [72], is required before any recommendations can be given about probiotic administration for asthma prevention.

### Vitamin D

Recently, Litonjua and Weiss hypothesized that vitamin D deficiency can increase the incidence of asthma in young children [73,74]. This idea stemmed from the discovery that the vitamin D receptor gene was associated with asthma [75]. (Albeit, more genetic work is necessary to clarify this since vitamin D receptor knockout mice do not develop the murine model for asthma [76].) Vitamin D does not occur naturally in humans and is acquired through supplements and exposure to sunlight. The rise of asthma in westernized countries may be linked to the fact that people spend much more time indoors and away from sunlight. Furthermore, vitamin D has significant immunomodulatory functions through control of T regulatory cells, which modulate levels of CD4+ helper T cells. Vitamin D receptors have been identified in various immune cells from T cells to dendritic cells that have a potential role in asthma pathogenesis.

Observational studies in the United States and the UK have reported that maternal intake of vitamin D during pregnancy was associated with lung function, suggesting that increased vitamin D in maternal diet may reduce risk of wheeze and other symptoms of asthma [77,78]. As with other hypotheses, supplementation studies are necessary, especially in pregnancy.

### Maternal diet hypothesis

Extending the "thrifty phenotype hypothesis" by Barker et al [79,80], maternal nutrition has been recognized as a potential (and potent) factor in the development of the fetal airway and immune system. Nutrients during pregnancy may affect T helper cell differentiation toward a Th2 bias through cytokine regulation and promote normal airway formation in the fetus [3].

With the prospect that diet during pregnancy may be more important than at any other point in life, many nutrients such as antioxidants and lipids have been tested. In 2002, Devereux et al found that increased maternal intake of vitamin E was associated with decreased proliferation of cord blood mononuclear cells in response to allergens, suggesting a beneficial effect of maternal nutrition against atopy [81]. Two separate maternal antioxidant studies showed an inverse relationship of antioxidants vitamin E, vitamin C, and zinc with wheeze [82,83]. The selenium status of a cohort of two thousand pregnant mothers was also inversely associated with wheezing in the child [84], but this disappeared after the age of five years. While these results indicate a possible role of maternal intake of certain antioxidants, more studies are necessary to confirm this. Studying the effects of maternal PUFA intake has been sparser, largely tested through analysis of maternal fish consumption. One such study found that maternal oily fish consumption during pregnancy was protective for childhood asthma, particularly in children who have asthmatic mothers [85]. In keeping with many other diet studies, however, a longitudinal study of maternal consumption of various food types found no association between fish intake and asthma outcomes in children [86]. There was also no association between asthma and maternal consumption of foods such as vegetables, egg, and dairy. In contrast to the more specific antioxidant and vitamin D studies, the effect of broader food groups on asthma outcomes seems less significant [87].

There is an obvious need for more intervention studies on dietary supplementation using nutrients and factors that have potential to impact the intrauterine environment and fetal immune and lung development [88]. Further understanding of dietary immunomodulation of the pregnant uterus is necessary [41]. With exciting developments elucidating the relationship between the in utero environment and subsequent onset of complex diseases, there is further motivation to explore the impact of diet on fetal development and risk of asthma.

## Conclusion: the road ahead

Asthma is complex: comprised of a heterogeneous variety of diseases, initiated by disparate genetic and environmental factors, and unified by common symptoms such as airway constriction and wheeze [89]. Diet could modulate epigenetics, intestinal microbiota, physiological development, airway remodeling, and immune maturation – factors highly relevant to the etiology of asthma. Yet the literature on diet and asthma is "fragmentary and hard to summarize in a systematic way and difficulties with many small studies leave unexplained contradictions in the literature" [10].

Such complexity makes for a daunting task of identifying pathways for future intervention. Evidence for nutrient supplementation after early childhood to support any primary prevention is weak. A greater understanding of maternal diet is necessary, particularly for antioxidants and vitamin D, perhaps by supplementing pregnant mothers with vitamin D and following their children through childhood [73]. Additionally, mechanistic studies are needed through gene expression and association studies. Explaining the downstream effects of vitamin D on infant physiology and immunology is crucial to vetting vitamin D as a possible intervention. One novel approach may be through genetic epidemiology using DNA collected from cohorts to analyze the effect of a modifiable factor by measuring variations in relevant genes [90]. Lastly, more extensive animal studies are necessary. There have been many diet-related studies using murine models of asthma. Admittedly, such models are relatively weak. Nevertheless, discoveries in a controlled animal model environment have advantages over the epidemiological approach in pursuing specific modalities [28,91,92].

Historically, studies have started from a population level formed from trends seen at the macro level with molecular mechanisms generally analyzed afterwards. With vitamin D [73] and maternal diet [3,80], there is a subtle but important difference in approach: mechanistic hypotheses at the micro level are now being expanded into larger clinical and population-based studies. Though it is still too early to determine if such an approach is beneficial, early indications are promising.

On the road ahead, if hypotheses are to be derived from the micro level, there is need for more collaboration amongst various groups from epidemiologists to geneticists to immunologists. As we look back and move forwards, a multidisciplinary approach is increasingly necessary to understand the complexity of dietary factors and asthma.

## Competing interests

The authors declare that they have no competing interests.

## Authors' contributions

J-HK undertook the literature review and drafted the manuscript. IA and PE conceived of the review, advised on strategy of the literature search and helped to draft the manuscript. All authors read and approved the final manuscript.

## Authors' information

IA chairs the International Study of Asthma and Allergies in Childhood (ISAAC). PE is a member of the ISAAC Steering Committee. IA and PE were lead authors on the dietary analysis of ISAAC Phase One. J-HK is a Harvard pre-medical student, who undertook this work during a Weissman Fellowship from the Harvard University.
